# Quality of Life after Post-Prostatectomy Intensity Modulated Radiation Therapy: Pelvic Nodal Irradiation Is Not Associated with Worse Bladder, Bowel, or Sexual Outcomes

**DOI:** 10.1371/journal.pone.0141639

**Published:** 2015-10-29

**Authors:** James M. Melotek, Chuanhong Liao, Stanley L. Liauw

**Affiliations:** 1 Department of Radiation and Cellular Oncology, University of Chicago, Chicago, Illinois, United States of America; 2 Department of Public Health Sciences, University of Chicago, Chicago, Illinois, United States of America; Sun Yat-sen University, CHINA

## Abstract

**Background:**

Limited data exist regarding toxicity and quality of life (QOL) after post-prostatectomy intensity modulated radiation therapy (IMRT) and whether pelvic nodal RT influences these outcomes.

**Methods:**

118 men were treated with curative-intent RT after radical prostatectomy. 69 men (58%) received pelvic nodal RT. QOL data and physician-assigned toxicity were prospectively collected. Changes in QOL from baseline were assessed with Wilcoxon signed-rank tests and risk factors associated with each domain were identified with generalized estimating equation (GEE) models. Late freedom from (FF) toxicity was estimated by the Kaplan-Meier method and comparisons were tested using the log-rank test.

**Results:**

Urinary irritation/obstruction, bowel, and sexual domain scores declined at 2 months (all *P* ≤ 0.01) but were no different than baseline at subsequent visits through 4 years of follow-up. At 4 years, FF grade 2+ GI toxicity was 90% and FF grade 2+ GU toxicity was 89%. On GEE analysis, pelvic nodal RT was associated with decreased bowel function (*P* = 0.09) and sexual function (*P* = 0.01). On multivariate analysis, however, there was no significant association with either decreased bowel *(P* = 0.31) or sexual (*P* = 0.84) function. There was also no association with either FF grade 2+ GI toxicity (*P* = 0.24) or grade 2+ GU toxicity (*P* = 0.51).

**Conclusions:**

Receipt of pelvic nodal RT was not associated with inferior QOL or toxicity compared to prostate bed alone RT. For the entire cohort, RT was associated with only temporary declines in patient-reported urinary, bowel, or sexual QOL.

## Introduction

Prostate cancer is the most common cancer in American men with 233,000 estimated new cases in 2014 [1]. Among men who receive treatment for localized prostate cancer, the majority undergo radical prostatectomy (RP) [2]. However, with certain high risk features, the risk of biochemical failure exceeds 50%. Radiation therapy (RT) after RP may be offered adjuvantly for adverse pathologic features [3–5] or for salvage after biochemical failure [6,7].

Notably, long term follow-up after post-prostatectomy radiation therapy (PPRT) suggests a moderate risk of relapse, even after a period of initial biochemical control. Although the role for intensification of PPRT by inclusion of the pelvic nodes has not been established prospectively, retrospective data demonstrate increased biochemical control in certain subsets of men [8,9]. However, the use of pelvic nodal RT in the post-operative setting may be tempered by concerns for increased toxicity. The primary goal of this study was to describe the influence of pelvic nodal RT on late toxicity and patient-reported quality of life (QOL) using a modern treatment approach including image guided, intensity modulated radiation therapy (IMRT). A secondary goal was to characterize QOL outcomes for the entire cohort of men treated with PPRT for use as a tool to guide patient expectations.

## Methods

Between 2006 and 2012, 122 men consecutively treated with PPRT were identified from a prospectively collected database. This study was approved by the University of Chicago Institutional Review Board (IRB). The majority of men in this study provided written informed consent during ongoing follow-up visits. Waiver of informed consent due to the retrospective nature of this work was obtained for all other patients in whom follow-up was not ongoing and therefore written informed consent could not be obtained in a practical fashion. The University of Chicago IRB approved both the written informed consent procedure and waiver of informed consent in patients who could not provide written informed consent. All men were treated with curative intent and had no known metastatic disease at the time of RT. Four men who had fewer than two QOL assessments were excluded from the study, leaving 118 men in the study cohort. The majority of men (87%) received salvage RT for rising PSA after RP. Median time from RP to RT was 18 months (range 3–135) and median pre-RT PSA was 0.24 (range 0–6.7). Androgen deprivation therapy (ADT) was given to 66 men (56%) and was typically a combination of an anti-androgen and a luteinizing hormone-releasing hormone agonist. Hormone therapy could have been given prior to the start of RT though no men in the study were castrate-resistant. QOL measures were assessed using the Expanded Prostate Cancer Index Composite (EPIC-26) by patients at baseline (and prior to any neoadjuvant ADT), at 2-months after completing treatment, and at subsequent biannual follow-up visits. Patients who completed QOL surveys at follow-up but not at the time of consultation were included in the analysis to assess changes in QOL over time after completion of RT. Physician-rated toxicity scores (CTC v4) for gastrointestinal (GI) and genitourinary (GU) domains were also assigned prospectively at each follow-up visit.

All patients underwent computed tomography (CT) simulation in the supine position with upper and lower alpha cradles for custom immobilization. The bladder was drained and introduced with 120 cc of saline; patients were instructed to maintain a comfortably full bladder at the time of each treatment. IMRT was used for both prostate bed alone RT and whole pelvic RT. The prostate bed was contoured according to published guidelines [10] with 0.6–0.9 cm anisotropic expansions to PTV, or a uniform 5 mm expansion if surgical clips were used for image guidance [11]. Pelvic lymph node contours were defined according to Radiation Therapy Oncology Group guidelines with 7–8 mm expansions around pelvic blood vessels to clinical target volume and additional 7–8 mm expansions to planning target volume (PTV). Patients receiving prostate bed RT were treated to a median dose of 68 Gy (interquartile range 66–68 Gy); patients in the pelvic nodal RT group were treated to a median dose of 50.4 Gy to the pelvic nodes and 68.4 Gy to the prostate bed (interquartile range 66.6–68.4 Gy). Treatment planning goals prioritizing PTV coverage and rectal, bladder, penile bulb, and femoral head sparing were used. Greater than 99% of the PTV was covered by the 95% isodose line with no volume receiving greater than 110% of the prescription dose. The volume of rectum and bladder receiving 70 Gy, 65 Gy, and 40 Gy were limited to 20%, 40%, and 80% and 30%, 60%, and 80%, respectively. The volume of penile bulb and femoral heads receiving 50 Gy were limited to 50% and 10%, respectively. Image guidance for setup verification was performed with cone beam CT on the first and second treatment days to ensure proper soft tissue alignment as well as daily kilovoltage imaging aligning to bony anatomy or to surgical clips in the prostate bed when present.

Differences in baseline patient characteristics and treatment variables between the prostate bed alone and pelvic nodal RT groups were assessed by t-test or Mann-Whitney test, depending on the distributions for continuous variables, and by chi-square or Fisher’s exact test for categorical variables. Wilcoxon signed-rank tests were used to assess the changes in QOL measures from baseline at 2-, 6-, 12-, 18-, 24-, 36-, and 48-month follow-up. In the event that data from the 24-, 36-, or 48-month follow-up was missing but was available for the subsequent 30-, 42-, or 54-month follow-up, respectively, the QOL survey from the later time point was used. Patients who experienced biochemical recurrence after PPRT continued to follow-up in Radiation Oncology and complete QOL surveys. Generalized estimating equation (GEE) models were used to identify risk factors associated with each QOL domain over time, respectively. Models included the tested covariate and assessment time as independent variables, the baseline scores, and the interaction term between the covariate and time. Factors, including age, race, body-mass index (BMI), presence of diabetes mellitus (DM) as a comorbidity, current or prior tobacco use, androgen deprivation therapy (ADT) use, time from prostatectomy to RT, receipt of pelvic nodal RT, and RT dose were tested for an association with each global domain endpoint as part of GEE and multivariate analyses. Freedom from toxicity beyond 3 months was estimated by the Kaplan-Meier method and statistical comparisons were tested using the log-rank test.

## Results

**Fig 1:**
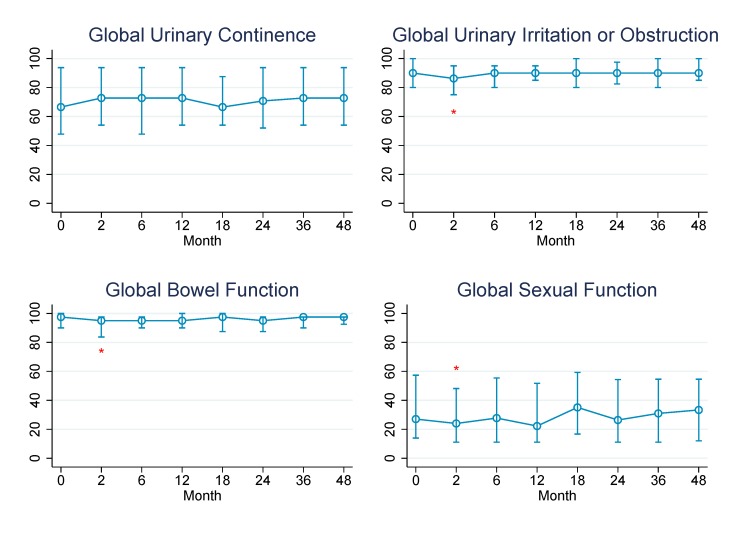
Changes in median QOL scores over time for the entire study cohort. Asterisks designate time points at which scores were significantly worse than baseline (*P* < 0.05 by Wilcoxon signed-rank test). Standard error bars represent interquartile range.

**Fig 2:**
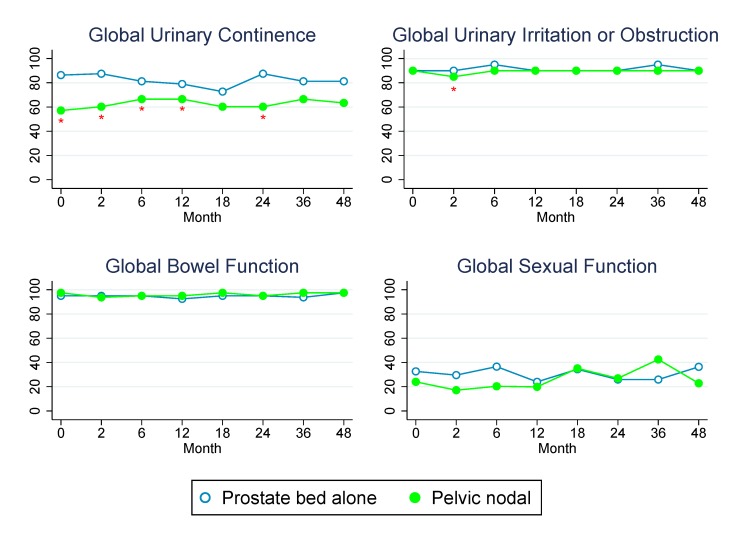
Changes in median QOL scores over time for patients treated with prostate bed versus pelvic nodal RT. Asterisks designate time points at which scores were significantly different between groups (*P* < 0.05 by Mann-Whitney test). Standard error bars represent interquartile range.

Baseline patient characteristics and treatment variables are presented in Table 1. Patients receiving pelvic nodal RT were significantly more likely to have a higher Gleason score, more advanced pathologic T stage, higher pre-RT PSA, lymph node positive disease, and receive ADT (all *P* ≤ 0.01). The percent of patients reporting specific levels of dysfunction or distress for each QOL domain at baseline and in follow-up is reported in Table 2. The median QOL scores over time for each domain are demonstrated graphically in Figs 1 and 2 and reported numerically below. GEE analyses for individual covariates and multivariate analyses were performed for each QOL domain and are shown in Table 3.

**Table 1:** Patient and treatment characteristics.

Variable	Prostate bed alone RT	Pelvic nodal RT	P value
	(N = 49)	(N = 69)	
Mean age, years (SD)	61.7 (6.9)	62.3 (6.5)	0.60
Race–no. (%)			0.47
White	32 (65.3)	51 (73.9)	
African American	14 (28.6)	13 (18.8)	
Other	3 (6.1)	5 (7.2)	
Median BMI (range)	29.8 (22.2–38.7)	28.5 (19.0–57.4)	0.57
Diabetes mellitus–no. (%)	3 (6.1)	13 (18.8)	0.06
Gleason score–no. (%)			<0.01
6	8 (16.3)	6 (8.7)	
7	35 (71.4)	34 (49.3)	
8	2 (4.1)	10 (14.5)	
9	4 (8.2)	19 (27.5)	
Pathologic T stage–no. (%)			0.01
pT2	21 (42.9)	14 (20.3)	
pT3a	20 (40.8)	32 (46.4)	
pT3b	7 (14.3)	23 (33.3)	
pT4	1 (2.0)	0 (0)	
Pathologic lymph node positive–no. (%)	0 (0)	14 (24.1)	<0.001
Median pre-RT PSA, ng/mL (range)	0.17 (0–0.87)	0.28 (0.07–6.7)	<0.01
ADT received–no. (%)	3 (6.1)	63 (91.3)	<0.001
Median ADT duration, months (range)	4 (3–5)	4 (1–28)	0.69
Median RT dose, Gy (IQR)	68 (66–68)	68.4 (66.6–68.4)	0.36
Median time from RP to RT, months (range)	16.5 (3–127)	19 (3–135)	0.80
Median follow-up, months (range)	38 (2–75)	25 (4–76)	0.17

**Table 2:** Percent of patients reporting specific levels of dysfunction or distress for each QOL domain.

Variable	Baseline	2 Mo	6 Mo	12 Mo	18 Mo	24 Mo	36 Mo	48 Mo
	(N = 102)	(N = 100)	(N = 98)	(N = 93)	(N = 69)	(N = 64)	(N = 50)	(N = 33)
**Urinary function**								
Irritation or obstruction								
Dysuria	2	3	0	0	1	0	0	0
Hematuria	0	1	1	2	0	2	2	0
Weak stream	5	1	2	0	1	5	0	0
Nocturia	10	17	9	13	7	9	13	13
Frequency	7	10	5	5	7	5	12	6
Incontinence								
Leaking ≥1 time per day	49	37	36	35	36	39	39	36
Frequent dribbling	13	12	8	12	10	14	16	12
Any pad use	39	30	33	28	38	35	24	27
Leaking problem	12	15	7	14	12	6	17	15
Overall urinary problem	10	13	8	11	9	6	12	3
**Bowel function**								
Urgency	3	7	3	2	6	5	2	3
Frequency	1	5	3	1	4	2	0	0
Fecal incontinence	1	0	0	1	1	3	0	3
Bloody stools	1	1	0	0	0	0	2	0
Rectal pain	3	5	1	0	1	3	0	0
Overall bowel problem	2	6	4	4	1	3	4	3
**Sexual function**								
Poor erections	69	70	64	67	58	65	63	66
Difficulty with orgasm	51	59	46	54	48	47	54	47
Erections not firm	69	78	68	77	65	77	70	76
Erections not reliable	63	71	65	67	58	72	64	66
Sexually active	33	32	33	30	38	28	37	37
Poor sexual function	62	68	63	65	60	66	56	72
Overall sexuality problem	44	31	42	42	24	27	33	28

**Table 3:** Factors associated with each QOL domain on multivariate analysis.

QOL domain	Independent variable	P value
**Urinary continence**	Age	0.73
	Race	
	*African American vs*. *White*	0.56
	*Other vs*. *White*	0.34
	BMI	0.02
	Diabetes mellitus	0.37
	Current tobacco use	0.11
	Prior tobacco use	0.72
	Pathologic T stage	0.49
	ADT use	0.62
	Time to RT	0.31
	Pelvic nodal RT	0.84
	RT dose	0.75
**Urinary irritation**	Age	0.31
	Race	
	*African American vs*. *White*	0.70
	*Other vs*. *White*	0.69
	BMI	0.02
	Diabetes mellitus	0.52
	Current tobacco use	0.10
	Prior tobacco use	0.49
	Pathologic T stage	1.00
	ADT use	0.41
	Time to RT	0.72
	Pelvic nodal RT	0.32
	RT dose	0.04
**Bowel function**	Age	0.49
	Race	
	*African American vs*. *White*	0.002
	*Other vs*. *White*	0.67
	BMI	0.04
	Diabetes mellitus	0.80
	Current tobacco use	0.13
	Prior tobacco use	0.96
	Pathologic T stage	0.73
	ADT use	0.56
	Time to RT	0.77
	Pelvic nodal RT	0.31
	RT dose	0.52
**Sexual function**	Age	0.05
	Race	
	*African American vs*. *White*	0.15
	*Other vs*. *White*	0.82
	BMI	0.22
	Diabetes mellitus	0.27
	Current tobacco use	0.48
	Prior tobacco use	0.32
	Pathologic T stage	0.42
	ADT use	0.14
	Time to RT	0.95
	Pelvic nodal RT	0.84
	RT dose	0.40

Global urinary continence was unchanged from baseline through 4 years of follow-up at all time points (*P* > 0.1) whereas global urinary irritation/obstruction was significantly worse than baseline (median, 90.0) at 2 month follow-up (86.3, *P* = 0.001) but no different than baseline at 6 month follow-up (90.0) and subsequent visits (*P* = 0.84). At 4 years, freedom from grade 1+ or 2+ GU toxicity was 57% and 89%, respectively. Higher BMI was associated with worse urinary continence scores on GEE *(P* = 0.02) and multivariate (*P* = 0.02) analyses; no other factors were associated with urinary continence, including pelvic nodal RT. However, patients treated with pelvic nodal RT did have lower baseline global urinary continence scores, which was likely related to the more aggressive resection for these men with higher risk disease as described in Table 1. Higher BMI was also associated with worse urinary irritation/obstruction on GEE *(P* = 0.02) and multivariate *(P* = 0.02) analyses. When BMI was tested as a categorical variable on multivariate analysis, obesity (BMI ≥ 30) was associated with worse global urinary continence (*P* = 0.04) but not urinary irritation or obstruction (*P* = 0.11). Additionally, higher RT dose was associated with worse urinary irritation/obstruction on GEE (*P* = 0.06) and multivariate analysis (*P* = 0.04). There was no association between receipt of pelvic RT and worse urinary continence or irritation/obstruction scores on GEE (*P* = 0.45, *P* = 0.89) or multivariate analyses (*P* = 0.84, *P* = 0.32). There was also no association between receipt of pelvic RT and freedom from grade 1+ (*P* = 0.39) or grade 2+ (*P* = 0.51) GU toxicity.

Global bowel function was significantly worse than baseline (97.5) at 2 month follow-up (95.0, *P* < 0.001) but was no different than baseline at 6 month follow-up (95.0) and subsequent visits (*P* > 0.1). At 4 years, freedom from grade 1+ or 2+ GI toxicity was 66% and 90%, respectively. African-American race was associated with worse global bowel function on GEE (*P* = 0.004) and multivariate analyses (*P* = 0.002), as was higher BMI (*P* = 0.04). Obesity, however, was not associated with worse bowel or rectal function on multivariate modeling (*P* = 0.27). On GEE analysis, receipt of hormone therapy (*P* = 0.07) and receipt of pelvic nodal RT (*P* = 0.09) were weakly associated with worse bowel function; however, on multivariate analysis, there was no association (*P* = 0.56, *P* = 0.31). There was also no association between receipt of pelvic RT and freedom from physician-assigned grade 1+ (*P* = 0.64) or grade 2+ (*P* = 0.24) GI toxicity.

Global sexual function was also significantly worse than baseline (27.0) at 2 month follow-up (24.0, *P* = 0.01) but was no different than baseline at 6 month follow-up (27.7) and subsequent visits (*P* > 0.1 at all time points). On GEE analysis, increasing age (*P* = 0.04), receipt of pelvic nodal RT (*P* = 0.01), receipt of hormone therapy (*P* = 0.01), and the presence of DM as a comorbidity (*P* = 0.04) were all associated with worse global sexual function. On multivariate analysis, however, only increasing age was associated with worse sexual function (*P* = 0.05).

## Discussion

In this cohort of men treated with PPRT who were prospectively evaluated with patient-reported QOL surveys and physician-rated toxicity, bladder, bowel, and sexual QOL remained stable through 4 years of follow-up, after only a transient decrease at 2 months. Additionally, the treatment of pelvic lymph nodes did not appear to negatively affect QOL or toxicity. Patients treated with pelvic nodal RT had higher risk disease, which likely contributed towards the relatively worse baseline urinary continence scores, but there was no disproportionate decline over time for men treated with pelvic nodal RT.

The negligible effect of pelvic nodal RT on toxicity in our series contrasts randomized data from the conventional (2D) RT era in the intact setting which demonstrated increased rates of late grade 2+ GU toxicity, and late grade 2 and 3+ GI toxicity with increasing field size for pelvic nodal coverage [12]. Our data are in line with other modern series which demonstrate reduced rates of acute [13] and late [14] GI toxicity with the use of IMRT in the post-prostatectomy setting. Our data also demonstrate stability with longer follow-up and more patients compared to an earlier analysis which showed no significant changes in urinary, bowel, or sexual QOL through 2 years of follow-up [15].

There are conflicting data regarding the toxicity of PPRT, perhaps related to differences in reporting and RT technique. In a patient-reported QOL analysis from the SWOG 8794 trial using a less detailed questionnaire than the EPIC-26, receipt of adjuvant RT was associated with worse bowel function through approximately 2 years after RT and worse urinary function through the reported 5 years of follow-up [16]. Toxicity data from the main trial also demonstrated high rates of urethral stricture and total urinary incontinence in patients receiving adjuvant RT, possibly because complications were published as crude percentages without regard to grade of toxicity or time of occurrence. In contrast, the EORTC 22911 trial demonstrated relatively low risks of severe acute and late toxicity with adjuvant RT. Additionally, a subset analysis demonstrated no difference in urinary continence as measured by pad weight between the group receiving adjuvant RT and the observation group [17]. In both of these trials, men were treated with conventional (2D) RT to 60–64 Gy. In a recent publication from Milan, receipt of dose-escalated adjuvant RT to a median dose of 70.2 Gy using 3D-conformal technique seemed to have a detrimental effect on recovery of urinary continence after RP [18]. In our series of mostly salvage PPRT to a median dose of 68 Gy using image-guided IMRT, global urinary continence scores demonstrated stability from baseline through 4 years of follow-up. Though global urinary irritation/obstruction, bowel function, and sexual function scores declined at 2 month follow-up, there was no difference from baseline on subsequent visits through 4 years of follow-up. Notably, RT dose was associated with urinary irritation/obstruction on multivariate modeling. It is possible that either the use of IMRT, the slightly lower dose prescribed in our series, or the generally delayed timing of RT mitigated the detrimental effect of PPRT on urinary function described in the Milan series.

It is interesting that higher BMI was associated with worse global urinary continence, urinary irritation/obstruction, and bowel or rectal function. Surgical series have demonstrated a similar association between obesity and delayed, less complete recovery of urinary [19,20] and bowel [21] function after RP, possibly due to the negative effect of obesity on the pelvic floor [22]. African-American race was also associated with worse global bowel or rectal function. Other series have similarly demonstrated worse urinary and bowel function in African-American men compared with white men after treatment of localized prostate cancer [23]; however, the small size of our series precludes us from drawing any firm conclusions from this result.

Though this analysis of prospectively collected data is limited by small sample size, it is among the few published series examining QOL after PPRT. It is reassuring to see no declines in QOL except for a transient decrease at 2 month follow-up, but it is theoretically possible that QOL scores could have continued to improve from baseline in the absence of PPRT. Given the median time from RP to RT in our series was 18 months, it is likely that bowel and bladder recovery of most patients had plateaued based on previously published QOL data after RP [24].

The benefit of pelvic nodal radiation for subsets of men in the post-prostatectomy setting has been suggested in retrospective series but awaits prospective validation. This is currently being investigated in RTOG 0534, which randomizes patients with rising PSA after RP to receive either prostate bed radiation with or without pelvic nodal RT. Until these data become available, our current institutional practice is to consider inclusion of the pelvic nodes in combination with ADT for select men with high-risk features. Although we report non-randomized, single-institution data, these results provide reassurance that intensified therapy may not significantly increase the risks of toxicity. Our patient reported outcomes data from a consecutively treated cohort can be useful to guide the expectation of men who are considering post-operative RT with modern radiation techniques.
